# Estimation of US Children’s Educational Attainment and Years of Life Lost Associated With Primary School Closures During the Coronavirus Disease 2019 Pandemic

**DOI:** 10.1001/jamanetworkopen.2020.28786

**Published:** 2020-11-12

**Authors:** Dimitri A. Christakis, Wil Van Cleve, Frederick J. Zimmerman

**Affiliations:** 1 Department of Pediatrics, Seattle Children’s Research Institute, University of Washington, Seattle; 2 Department of Anesthesiology and Pain Medicine, University of Washington, Seattle; 3 Department of Health Policy & Management, Center for Health Advancement, Fielding School of Public Health, University of California, Los Angeles, Los Angeles, California

## Abstract

**Importance:**

United States primary school closures during the 2020 coronavirus disease 2019 (COVID-19) pandemic affected millions of children, with little understanding of the potential health outcomes associated with educational disruption.

**Objective:**

To estimate the potential years of life lost (YLL) associated with the COVID-19 pandemic conditioned on primary schools being closed or remaining open.

**Design, Setting, and Participants:**

This decision analytical model estimated the association between school closures and reduced educational attainment and the association between reduced educational attainment and life expectancy using publicly available data sources, including data for 2020 from the US Centers for Disease Control and Prevention, the US Social Security Administration, and the US Census Bureau. Direct COVID-19 mortality and potential increases in mortality that might have resulted if school opening led to increased transmission of COVID-19 were also estimated.

**Main Outcomes and Measures:**

Years of life lost.

**Results:**

A total of 24.2 million children aged 5 to 11 years attended public schools that were closed during the 2020 pandemic, losing a median of 54 (interquartile range, 48-62.5) days of instruction. Missed instruction was associated with a mean loss of 0.31 (95% credible interval [CI], 0.10-0.65) years of final educational attainment for boys and 0.21 (95% CI, 0.06-0.46) years for girls. Summed across the population, an estimated 5.53 million (95% CI, 1.88-10.80) YLL may be associated with school closures. The Centers for Disease Control and Prevention reported a total of 88 241 US deaths from COVID-19 through the end of May 2020, with an estimated 1.50 million (95% CI, 1.23-1.85 million) YLL as a result. Had schools remained open, 1.47 million (95% credible interval, 0.45-2.59) additional YLL could have been expected as a result, based on results of studies associating school closure with decreased pandemic spread. Comparing the full distributions of estimated YLL under both “schools open” and “schools closed” conditions, the analysis observed a 98.1% probability that school opening would have been associated with a lower total YLL than school closure.

**Conclusions and Relevance:**

In this decision analytical model of years of life potentially lost under differing conditions of school closure, the analysis favored schools remaining open. Future decisions regarding school closures during the pandemic should consider the association between educational disruption and decreased expected lifespan and give greater weight to the potential outcomes of school closure on children’s health.

## Key Points

**Question:**

Based on the current understanding of the associations between school disruption and decreased educational attainment and between decreased educational attainment and lower life expectancy, is it possible to estimate the association between school closure during the coronavirus disease 2019 pandemic and decreased life expectancy of publicly educated primary school–aged children in the United States?

**Findings:**

This decision analytical model found that missed instruction during 2020 could be associated with an estimated 5.53 million years of life lost. This loss in life expectancy was likely to be greater than would have been observed if leaving primary schools open had led to an expansion of the first wave of the pandemic.

**Meaning:**

These findings suggest that the decision to close US public primary schools in the early months of 2020 may be associated with a decrease in life expectancy for US children.

## Teaser

This decision analytical model estimates the potential years of life lost among US primary school–aged children associated with school closures during the coronavirus disease 2019 pandemic.

## Introduction

In early 2020, school closures were widely instituted across the United States as a coronavirus disease 2019 (COVID-19) containment strategy. The rationale for closures was 2-fold. First, at least initially, the risks that the virus posed to children were unclear but worthy of precaution. Second, it was assumed that children might represent important vectors for disease spread even if they were themselves unaffected or asymptomatic. Both of these considerations appeared to justify the harm of missed education in order to minimize the population-level risk of disease. In the ensuing months, data have emerged indicating that COVID-19 infection poses significantly less direct risk to children to adults.^1,2^ While the scientific evidence on transmission of SARS-CoV-2 by children remains in flux, recent studies indicate that young children (<10 years) appear less likely to serve as vectors for COVID-19 transmission.^3,4^ Although the risks of keeping schools open drove decisions made in the early phases of the pandemic, the probable harm to children associated with school closure were less explicitly discussed.^5^ The public debate has pitted “school closures” against “lives saved,” or the education of children against the health of the community. Presenting the tradeoffs in this way obscures the very real health consequences of interrupted education.

These consequences are especially dire for young children. There is little reason to believe that virtual learning environments can be effective for primary school–aged children. A meta-analysis^6^ of 99 experimental studies included only 5 conducted in school-aged children, and they were primarily in fifth through eighth grade. The meta-analysis concluded that “the mean effect size [for online learning] is not significant for the seven contrasts involving K-12 students.”^6^^(pXV)^ That so few studies have even been conducted in this age group is also telling. A recent study comparing Indiana children in grades 3 through 8 who switched from brick-and-mortar to virtual schooling “experienced large, negative effects in math and [English/language arts] that were sustained across time”.^7^^(p170)^ Sal Khan, a widely respected innovator in the field of distance learning, reported that distance learning approaches do not work for younger students.^8^ Furthermore, it is not clear how much access to remote instruction primary school–aged children actually received during the spring of 2020. For example, in its March 2020 guidelines for districts, the Illinois Department of Education recommended that primary school children have a maximum of 60 to 120 minutes per day in remote learning, representing a fraction of a regular school day.^9^ In 2 national surveys, teachers of all grades reported that only 60% of their students were regularly engaging in distance learning at all, and only 27% of teachers took attendance.^10,11,12^ Accordingly, it is reasonable to infer that primary school–aged children received minimal meaningful instruction beyond what is being delivered by their parents or other caregivers at home.^13^ It is not surprising, then, that the National Academies of Science, Engineering, and Medicine’s report on school openings^13^ concluded that districts should make returning primary school children to in-person classes a priority.

Evidence suggests that missing school has adverse effects on eventual educational attainment. A longitudinal study of teacher strikes in Argentina revealed that disrupted schooling lowered graduation rates, total educational attainment, and subsequent income.^14^ An educational reform in Belgium differentially affected Flemish-speaking and French-speaking parts of the country and resulted in strikes of approximately 60 days in the French-speaking part of the country against none in the Flemish-speaking part. Using this natural experiment in a difference-in-difference framework, economists estimated the long-term effects of these strikes on educational attainment to be a 5.8% reduction in total years of educational attainment, a somewhat larger effect than that identified in Argentina.^15^ Prolonged strike studies in the United States and Canada are lacking, but even short-term strikes were found to result in diminished test scores.^16,17^ One US report^18^ found that the single best predictor of high-school graduation was fourth-grade reading test scores: 23% of children who are not reading at grade level by the end of third grade will not graduate high school, compared with 9% of those who are. The risks are even greater for low-income Black or Hispanic students: 33% of those not reading at grade level will not graduate from high school. These educational impairments are in turn consequential for mortality: the quality and quantity of education received today have considerable effects on life expectancy.^19,20,21,22^

The American Academy of Pediatrics’ policy statement on school reopening suggested that science drive decision-making.^23^ Doing so requires a better-informed estimate of the tradeoffs being considered. The primary objective of this study was to model the expected years of life lost (YLL) in association with primary school closures in early 2020 and to compare them to potential YLL had schools remained open.

## Methods

This decision analytical model consisted of an analysis of publicly available data. Our analysis and report follows the Consolidated Health Economic Evaluation Reporting Standards (CHEERS) reporting guideline.^24^ As an analysis of publicly available data, our institutions considered this work exempt from institutional review board review.

**Figure 1.**
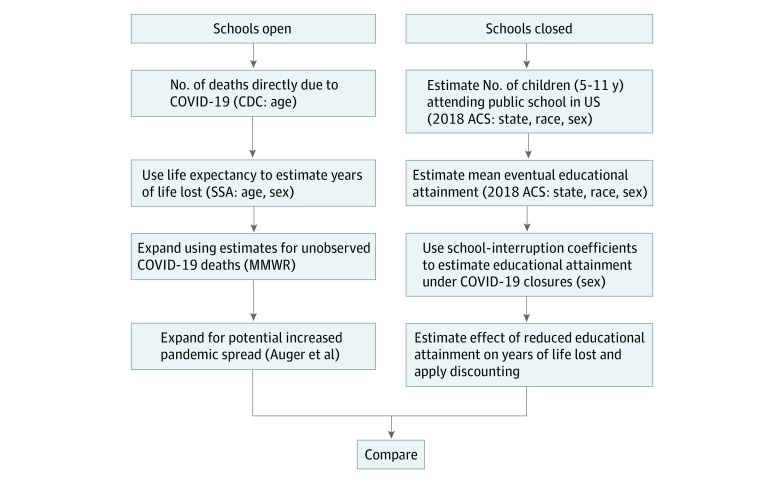
Analysis Overview ACS indicates US Census Bureau American Community Survey; CDC, US Centers for Disease Control and Prevention; COVID-19, coronavirus disease 2019; MMWR, *Morbidity and Mortality Weekly Report*; and SSA, US Social Security Administration.

Our goal was to compare the number of expected YLL due to COVID-19 in the United States under 2 different scenarios (Figure 1). Scenario 1, observed from January 1 to May 30, 2020, consisted of the closing of primary schools during the early phase of the COVID-19 pandemic in the United States, resulting in expected YLL from 2 primary pools: premature deaths from COVID-19 and shortened life expectancy due to decreased educational attainment among children. Scenario 2 was unobserved and was based on a counterfactual decision to allow primary schools to remain open, with a potentially increased number of deaths and years of life directly lost due to COVID-19 if school opening led to increased pandemic spread.

### Statistical Analysis

#### Scenario 1 Estimation

To estimate the YLL due to COVID-19 under conditions of school closure, we required an estimate of direct mortality from COVID-19 during the early phase of the pandemic (see Scenario 2 Estimation) and an estimate of the YLL associated with primary school closure. This latter estimate was constructed in 2 stages: we first estimated the association between school closures and educational attainment, and then we estimated the association between reduced educational attainment and mortality risk. For stage 1, we used the results of a carefully constructed econometric analysis of quasi-random school closures due to teachers’ strikes in Argentina. These results—which use within-province differences in strike exposure across birth cohorts and within-cohort differences in strike exposure across provinces as sources of exogenous variation—provide a reasonable estimate of the association between missed education during primary school and total years of educational attainment.^14^ This analysis suggests that 10 days of missed school is associated with a reduction in final educational attainment of 0.0262 (SE, 0.0064) years for boys and 0.0217 years (SE, 0.0062) years for girls.

To apply this analysis to the US, we used the 2018 1-year estimates from the US Census Bureau American Community Survey (ACS) and counted all children between the ages of 5 and 11 (inclusive) who were enrolled in public school. Because life expectancy varies by race/ethnicity, we categorized children as White, Black, Hispanic, or Other. Children in the Other category were assigned the average life expectancy (nonconditioned on race/ethnicity), whereas other races/ethnicities were assigned specific life expectancies based on life tables for these groups available from the US Centers for Disease Control and Prevention (CDC).^25^ State-specific missed days of education were estimated by reviewing published school closure orders and school calendars from school districts in the capital of each state and counting weekdays only. We assumed that final educational attainment reported by respondents aged 25 to 29 years in the 2018 ACS (again stratified by sex, race/ethnicity, and state) would be representative of eventual attainment of children attending primary school in 2020.

Prior research in economics has estimated the association between decreased educational attainment and life expectancy using a variety of causal inference techniques.^21,22,26,27,28,29,30^ Using the method described in the eAppendix in the Supplement, we estimated a weighted average effect of these estimates, which suggested that each additional year of education attained is associated with a relative risk of 0.75 (95% CI, 0.60-0.90) of annual mortality. This relative risk was then applied to the most recent life table published by the CDC^25^ to obtain YLL estimates across the life course. Uncertainty estimates were generated by a Monte Carlo simulation, treating the associations between school closure and education and between education and life expectancy as truncated normal and normally distributed variables, respectively.

In cost-benefit analyses, it is customary to apply a discount factor to costs and benefits that occur in the future so that they can reasonably be compared to current values. Although discounting of future costs is reasonable, how and even whether to discount future health benefits is controversial, with no clear consensus in the literature.^24,25,26,27^ Yet because the association of missed education with YLL of today’s children occurs so far in the future, discounting is consequential to the analysis. We therefore summarized the YLL associated with decreased educational attainment under 3 different annual discounting scenarios: no discount, 0.5%, and 3%, with the understanding that the choice of the appropriate discount rate represents a value judgment (see Discussion).^31,32,33,34^ The simulation results are reported for the nation as a whole in terms of total estimated YLL.

#### Scenario 2 Estimation

Estimation of YLL under unobserved conditions (ie, US primary schools being left open with a potential for increased spread of COVID-19) necessarily required explicit modeling of a range of potential scenarios. In order to model parameter uncertainty, we again performed a Monte Carlo simulation, this time using a Program Evaluation Research Task (PERT) distribution.^35^ The PERT distribution is a modification of the beta distribution with 2 favorable characteristics for this type of analysis. First, it allows for explicit specification of the minimum, maximum, and most likely (modal) values of the parameter. Second, it assigns greater probability weight to the modal value (approximately 4-fold greater) while still incorporating the minimum and maximum values into the resultant distribution. Like all beta distributions, it is positive-bounded.

We used CDC data on patient ages and numbers of COVID-19 deaths observed in early 2020 as the source of our mortality estimates and merged this data with sex-specific actuarial life tables published by the US Social Security Administration to estimate the lost years of life expectancy incurred by deaths in each age group, assuming that deaths occurred in the middle of the 10-year span and that 85 was the maximum age of death.^25^ There is broad acceptance that CDC mortality estimates for COVID-19 are an underestimate; we corrected for this bias by modeling a multiplicative ratio of YLL using a PERT distribution. We defined the minimum ratio as 1 (excess deaths being unlikely to be negative), used CDC estimates of excess mortality observed in New York state in early 2020 to define a modal ratio of 1.22, and doubled the modal estimate as our maximum.^36^ This method provided a distributional estimate of US YLL directly due to COVID-19 observed in 2020.

The next question we considered was how many deaths might have occurred (and how many YLL would have resulted) had primary schools remained open. Again, substantial uncertainty exists regarding the degree to which school closures affect the spread of COVID-19, with some authors^4^ suggesting that young children contribute very little to the spread of SARS-CoV-2 and others suggesting that school closures might play a substantial role in the pandemic’s spread.^37^ Auger and colleagues^37^ recently published an estimate that school closures may have prevented 40 600 US deaths during the early phase of the pandemic, with a mortality rate of 19.4 deaths per 100 000 population under school opening and 6.8 per 100 000 under school closure. In contrast, Courtemanche and colleagues^38^ estimated a nonsignificant daily decrease in mortality associated with school opening. Using a PERT distribution, we used the Auger mortality ratio (2.85) as our maximum estimate. Given the belief on the part of some investigators that young children do not measurably influence disease spread, we used a mortality ratio of 1 as our minimum estimate. To define a plausible modal estimate, we used the average of the Auger and Courtemanche mortality ratios, using a baseline schools-closed mortality rate in the Courtemanche estimate (which was not provided) equal to that in Auger. This yielded a modal mortality ratio of 1.93.

We summarized our data with 95% credible intervals (95% CIs) drawn from our Monte Carlo estimates, constructed distributional summary plots, and finally estimated the probability that YLL associated with COVID-19 in 2020 were greater under primary school closure than had schools remained open by enumerating the proportion of Monte Carlo draws where this event was observed. All statistical analyses and graphs were constructed using R, version 3.6.2 (R Foundation).

## Results

Using the 2018 ACS, we estimated that approximately 24.2 million children aged 5 to 11 years attended public schools that were closed during the 2020 pandemic (11.4 million White, 4.3 million Hispanic, 3.6 million Black, and 4.9 million Other). Across all US states, we estimated that public schools were closed for a median 54.0 days as a result of COVID-19 (IQR, 48-62.5 days). The mean (SD) final years of educational attainment reported by 25- to 29-year-old respondents in the 2018 ACS was 13.7 (2.1). We estimated that primary school–aged boys could lose, approximately 0.15 (95% credible interval, 0.08-0.35) final years of education as a result of school closure (, whereas girls could lose 0.12 (95% CI, 0.05-0.19) years. These losses in education were associated with a mean loss of life of 0.31 (95% CI, 0.10-0.65) years for boys and 0.21 (95% CI, 0.06-0.46) for girls. Undiscounted, these estimated losses in life due to missed education summed to a median estimate of 5.53 million (95% CI, 1.88-10.80) YLL. Under conditions of 0.5% annual discounting, YLL were estimated at 4.39 (95% CI, 1.50-8.60) million. Under 3% annual discounting, YLL were estimated at 1.52 (95% CI, 0.52-2.99) million.

The CDC reported a total of 88 241 US deaths from COVID-19 through May 30, 2020. Using Social Security Administration life tables, we estimated that these deaths resulted in 1 146 136 YLL (Table). Adjusting for potential undercounting of COVID-19 deaths led us to estimate that US deaths due to COVID-19 in early 2020 generated 1.50 million (95% CI, 1.23-1.85 million) YLL. Applying the counterfactual procedure described in our methods, we further estimated that had primary schools remained open and, in doing so, permitted the pandemic to spread, we might have observed a total of 2.97 million (95% credible interval, 1.88-4.30 million) YLL, with 1.47 million (95% credible interval, 0.45-2.59 million) YLL associated with schools remaining open.

**Table.** Years of Life Lost Due to COVID-19 Deaths Through June 3, 2020

Age, y	Male				Female			
	COVID-19 deaths	Death probability	Life expectancy	YLL, thousands	COVID-19 deaths	Death probability	Life expectancy	YLL, thousands
0	3	0.0063	76.0	0.22	2	0.0052	81.0	0.16
1	1	0.0004	75.5	0.08	2	0.0003	80.4	0.16
10	10	0.0001	66.5	0.67	2	0.0001	71.5	0.14
20	68	0.0011	56.9	3.87	38	0.0004	61.6	2.34
30	396	0.0019	47.7	18.87	187	0.0008	52.0	9.71
40	1094	0.0025	38.6	42.18	430	0.0014	42.5	18.26
50	3009	0.0050	29.7	89.28	1229	0.0031	33.2	40.83
60	6988	0.0115	21.6	150.80	3598	0.0069	24.6	88.37
70	11 479	0.0229	14.4	165.18	6881	0.0153	16.5	113.81
80	12 961	0.0582	8.3	107.32	10 650	0.0428	9.7	103.09
85	11 566	0.0979	5.9	68.12	17 647	0.0743	7.0	122.65

Abbreviations: COVID-19, coronavirus disease 2019; YLL, years of life lost.

**Figure 2.**
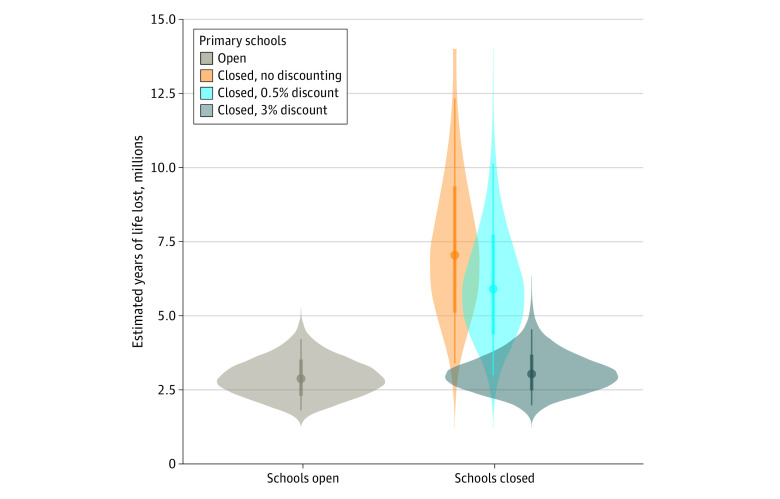
Estimated Years of Life Lost Center dots indicate medians; thin lines, 91% probability intervals; thick lines, 50% probability intervals; and shaded areas, probability density of the estimated years of life lost under each condition.

Finally, we compared the YLL in each scenario: the scenario in which the United States experienced deaths due to COVID-19 as well as decreased predicted life expectancy due to decreased educational attainment associated with school closures (ie, the path followed by the United States in early 2020 of primary schools closed) or an alternate, counterfactual scenario in which the country had left schools open, with a possible increase in transmission of COVID-19. The results of this analysis, including modifications of these estimates by discounting future deaths, are depicted in Figure 2. When comparing the distribution of probable YLL under these “primary schools open” and “primary schools closed” scenarios, we estimated a 98.1% probability that nondiscounted YLL would be greater under school closure than had schools remained open (96.6% under 0.5% discounting and 53.1% under 3% discounting).

## Discussion

In this decision analytical model, we estimated that there is a 98.1% probability that the decisions to close US primary schools in March of 2020 could be associated with more eventual YLL than would be observed if these schools had remained open, even if schools remaining open had led to a substantial increase in the rate of death observed during the early phase of the pandemic. Our analysis was based on the decreased life expectancy associated with a decrease in the mean years of educational attainment that can be expected to occur as a result of the disruption in schooling during a critical period of educational development. These potential associations of school closure with child health remain hidden at present, as the shortening of lifespans that today’s primary school children could experience would not manifest until long after the pandemic is over. The public discourse on reopening has focused on lives saved in the present and largely neglected the years of life that may be lost in the future because of decisions made today.

We believe that the debates over opening schools have often lacked nuance, portraying schools monolithically—as all opened or all closed—without regard for potential safeguards that could reduce transmission within schools. Furthermore, to our knowledge, very little discussion has considered the very different outcomes and costs associated with school closure for children at different ages and developmental stages. Based on the existing evidence regarding the limited role primary school–aged children play in transmission of COVID-19 and the heavy burden of decreased educational exposure on their health, we believe that restoring access to in-person primary school education should be an immediate national priority, even while the country awaits a vaccine.^4,39^

There are several ways in which our estimates of YLL are conservative. First, we focused only on interruptions to primary school. We did this because distance learning has been demonstrated to be largely ineffective for this age group, and there are well-controlled longitudinal data to model the association between primary school disruption and final educational attainment. Nevertheless, we believe it highly probable that school closures in early 2020 will also ultimately depress high school graduation rates (and therefore may influence lifespan) for older children as well. A recent McKinsey report^40^ estimated that 2% to 9% of high school students could drop out of school because of COVID-19–related school absences. These losses in educational attainment and resultant increased mortality are not included in our estimates. Second, we adjusted the number of COVID-19–related deaths in early 2020 to account for underreporting, thus favoring the schools-closed scenario. Third, we used a well constructed estimate of increased mortality associated with school opening to inform our estimate of potential increased pandemic spread associated with school opening. In fact, in a recent study, the net contribution of school closures to virus spread was found to be close to zero.^39^ Fourth, whereas other models have been criticized for their complexity and opacity, our model is simple and transparent.^41,42^ Finally, our estimates do not consider quality of life or disability associated with decreased educational attainment, which would be difficult to assess. However, it stands to reason that by not graduating high school, children may experience lower wages and the attendant detriments to quality of life for the duration of their lives.

### Limitations

Our study has limitations that we openly acknowledge. First, we based our estimates of decreased educational attainment associated with school closures on data from a single study performed in Argentina. Although we believe this study’s methods to be robust, the extent to which these data can be applied to a US population is unknown. Our results are reliant on the assumptions that the disruptions in Argentinian schooling observed during teacher strikes mimic those of school closures due to COVID-19 and that these disruptions will have similar effects on eventual educational attainment. We believe these assumptions are reasonable; we base this belief, in part, on a recent US study that confirms that falling behind in primary school has a significant negative association with rates of high school graduation.^18^ Students in the United States also have some access to distance learning (not present in the Argentinian example), and it can be argued that US children received at least some form of education during COVID-19–related closures. The effectiveness of distance learning for primary school–aged children is highly questionable. Moreover, in 28 states representing 48% of children in the United States, distance learning was not mandated, and many children received none.^11,43^ As for parental teaching, it is probable that Argentinian parents likewise provided some level of instruction to their children during the prolonged strikes. Third, the estimates of YLL directly due to COVID-19 in our study are dependent on the population prevalence of SARS-CoV-2 during that time. Since the end of May 2020, some communities have seen increases in disease prevalence, and a few have seen decreases. The association between school opening and population disease prevalence remains uncertain, and school opening could lead to greater transmission of disease among both adults and children. The most recently available data suggests that children younger than 10 years (consistent with the age we used in our model) transmit the virus considerably less readily than adults, suggesting that community prevalence may not be as important a factor in this population.^4^ Our analysis, however, presupposes that leaving schools open would be associated with a substantial increase in pandemic spread. Despite this obviously undesirable potential outcome, very likely borne to some extent by teachers and elderly relatives, the net YLL due to COVID-19 (at least during the early pandemic) appears to have favored schools remaining open.

Finally, our estimates are sensitive to the discount rate applied to future health outcomes. Discounting for conditions that affect young children is a matter of considerable controversy and represents the values that society places on children vs adults. Many argue against discounting of any kind, some authors argue for 0.5% annual discount rates, and others argue for 3% or even greater.^31,33,34^ As a matter of pure math, a discount rate of 3% heavily biases against any outcome that will occur in the distant future. In fact, any intervention that affects children over the entirety of their lifespans will be penalized by a high discount rate. Prior studies examining death from H1N1 influenza selected discount rates of zero.^44^ We presented 3 different rates to highlight that prior beliefs about future event value will influence policy changes. Our belief is that the future health outcomes of children deserve our full attention and consideration today and that a discount of 0 or 0.5% is most appropriate. However, our findings suggest that even under a 3% discount rate, the probability that life years were saved owing to school closures may essentially be a toss-up.

## Conclusions

This study has important implications. The results of this decision analytical model suggest that the attempt to save lives by closing schools may not have resulted in a net savings when considering the potential harms associated with this intervention. This lack of intergenerational health equity strikes us as unjust and deserves careful societal consideration. Another implication is that the losses being experienced among children are unlikely to be equitably distributed across the boundaries of gender, socioeconomic status, and race/ethnicity. Both Argentinian and US data suggest the influence of educational interruption is greater for low-income children and for boys, suggesting that the potential outcomes of school closures may be felt more substantively by vulnerable populations in the United States.^14,18^ Those outcomes are compounded by the fact that there was demonstrably less engagement in distance learning among low-income minority children: schools serving predominantly Black and Hispanic students reported that only 60% to 70% were participating in remote education on a regular basis, and only one-third were participating daily.^11,40,43^ Finally, to preserve intergenerational equity, the costs of future life years lost for young children today must be factored into decision-making regarding school openings and potential future closings. We believe that during the COVID-19 pandemic, the United States has extracted an enormous sacrifice from its youngest citizens to protect the health of its oldest. During a pandemic, this may well be an ethically defensible tradeoff, but only if resources are invested to reverse the potential damage to health and education that this strategy may inflict on a population with low visibility and high vulnerability.

## Notes

Supplement. **eAppendix.** Notes on Education-Mortality Link **eReferences.**
