# Computational Errors and Incorrect Analysis in Study on Primary School Closures

**DOI:** 10.1001/jamanetworkopen.2020.37247

**Published:** 2021-01-08

In the Original Investigation, “Estimation of US children’s Educational Attainment and Years of Life Lost Associated With Primary School Closures During the Coronavirus Disease 2019 Pandemic,”^1^ published on November 12, 2020, there were computational errors and an incorrect analysis used. Corrections to address these errors include the presentation of 2 estimates for potential years of life lost associated with school closure, using separate weighted relative risk estimates from US-based and European-based studies for the association between lost education and mortality. These corrections affect the Abstract; Methods, Results, and Discussion sections; Figure 2; and the Supplement. The authors have published a Comment with additional clarification and explanation of these corrections.^2^ The article has been corrected.^1^

## References

[1] Christakis DA, Van Cleve W, Zimmerman FJ Estimation of US children’s educational attainment and years of life lost associated with primary school closures during the coronavirus disease 2019 pandemic. JAMA Netw Open. 2020;3(11):e2028786. doi:10.1001/jamanetworkopen.2020.28786 33180132 PMC7662136

[2] Christakis DA Response to concerns, clarifications, and corrections. JAMA Netw Open. January 8, 2021. doi:10.1001/jamanetworkopen.2020.28786 33180132 PMC7662136

