# Supplementary material for: Estimation of US Children’s Educational Attainment and Years of Life Lost Associated With Primary School Closures During the Coronavirus Disease 2019 Pandemic
Source: JAMA Netw Open. 2020 Nov 12;3(11):e2028786. doi: 10.1001/jamanetworkopen.2020.28786 (PMC7662136; doi:10.1001/jamanetworkopen.2020.28786)
Supplement: Supplement. — eAppendix. Notes on Education-Mortality Link eReferences. [file jamanetwopen-e2028786-s001.pdf]

Supplemental Online Content

Christakis DA, Van Cleve W, Zimmerman FJ. Estimation of US children's educational attainment and years of life lost associated with primary school closures during the coronavirus disease 2019 pandemic. *JAMA Netw Open*. 2020;3(11):e2028786.
doi:10.1001/jamanetworkopen.2020.28786

eAppendix. Notes on Education-Mortality Link
eReferences.

This supplemental material has been provided by the authors to give readers additional information about their work.

© 2020 Christakis DA et al. *JAMA Network Open*.

eAppendix. Notes on Education-Mortality Link

Although there is a steep education gradient in all health outcomes, including mortality, it is unclear how much of this gradient is causal. We searched the peer-reviewed literature to find papers that attempted to isolate the causal effect of an additional year of educational attainment on mortality risk and that included enough detail about methods to calculate a relative risk reduction. We used the 7 studies meeting these criteria[1-7] to contribute to an overall estimate of the marginal effect of years of education on mortality risk. Studies were weighted by the inverse of the variance of the estimates, with US-based studies assigned double this value.

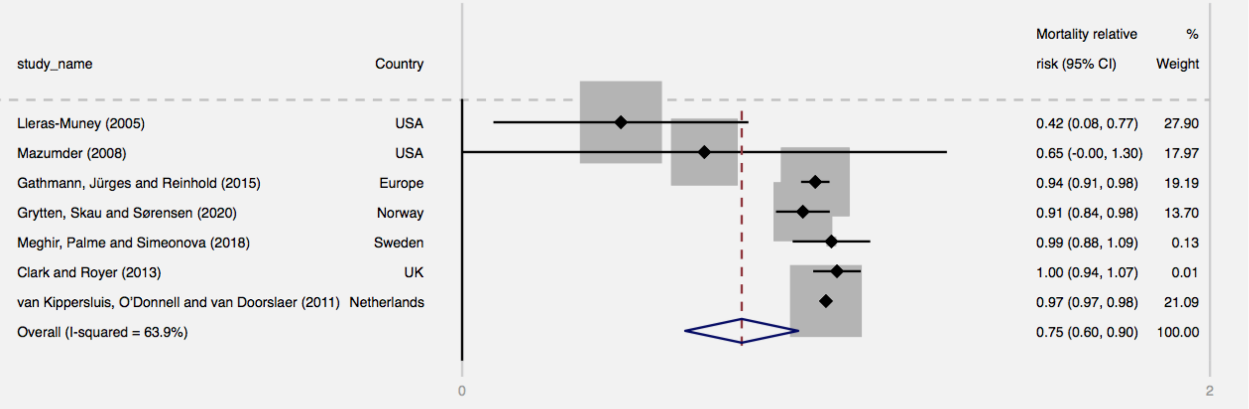

eReferences

1. Grytten J, Skau I, Sørensen R. Who dies early? Education, mortality and causes of death in Norway. *Social Science & Medicine*. 2020;245:112601.
2. Gathmann C, Jürges H, Reinhold S. Compulsory schooling reforms, education and mortality in twentieth century Europe. *Social Science & Medicine*. 2015;127:74-82.
3. Mazumder B. Does education improve health? A reexamination of the evidence from compulsory schooling laws. *Economic Perspectives*. 2008;32(2).
4. Lleras-Muney A. The Relationship Between Education and Adult Mortality in the United States. *The Review of Economic Studies*. 2005;72(1):189-221.
5. Meghir C, Palme M, Simeonova E. Education and Mortality: Evidence from a Social Experiment. *American Economic Journal: Applied Economics*. 2018;10(2):234-256.
6. Clark D, Roayer H. The effect of education on adult mortality and health: Evidence from Britain. *The American Economic Review*. 2013;103(6):2087-2120.
7. van Kippersluis H, O'Donnell O, van Doorslaer E. Long Run Returns to Education: Does Schooling Lead to an Extended Old Age? *J Hum Resour*. 2009;4:1-33.

© 2020 Christakis DA et al. *JAMA Network Open.*
